# Implementation of a fluid balance control strategy in critically ill patients: POINCARE-2 trial process evaluation

**DOI:** 10.1186/s12874-024-02288-1

**Published:** 2024-07-24

**Authors:** Marie Buzzi, Laetitia Ricci, Sébastien Gibot, Laurent Argaud, Julio Badie, Cédric Bruel, Claire Charpentier, Hervé Outin, Guillaume Louis, Alexandra Monnier, Jean-Pierre Quenot, Francis Schneider, Laetitia Minary, Nelly Agrinier

**Affiliations:** 1 CIC, Epidémiologie Clinique, CHRU-Nancy, INSERM, Université de Lorraine, Nancy, F-54000 France; 2 Université de Lorraine, Inserm, INSPIIRE, Nancy, F-54000 France; 3 Service de Réanimation Médicale, CHRU Nancy, Université de Lorraine, Nancy, F-54000 France; 4 Service de réanimation médicale, Hospices civils de Lyon, Hôpital Edouard Herriot, Lyon, F-69000 France; 5 Service de Réanimation médicale, Hôpital Nord Franche-Comté, Belfort, F-90015 France; 6 Service de réanimation polyvalente, Groupe hospitalier Paris Saint-Joseph, Paris, F-75000 France; 7 CHRU-Nancy, Service d’Anesthésie Réanimation chirurgicale, Université de Lorraine, Nancy, F-54000 France; 8 Service de Réanimation, CHI Poissy Saint-Germain, Poissy, F-78303 France; 9 Service de Réanimation polyvalente, CHR Metz-Thionville, Metz, F-57000 France; 10 Service de Réanimation médicale, CHRU Strasbourg, Nouvel Hôpital Civil, Strasbourg, F-67000 France; 11 Service de Médecine Intensive-Réanimation, CHU Dijon-Bourgogne, Dijon, F-21000 France; 12 Service de Médecine Intensive Réanimation, CHU Strasbourg, Hôpital de Hautepierre, INSERM U 1121, Strasbourg, F-67000 France; 13 CIC-EC, CHRU Nancy Hôpitaux de Brabois, 9 allée du Morvan, Vandoeuvre-lès-Nancy, 54500 France

**Keywords:** Process evaluation, Randomized controlled trial, Complex intervention, Intensive care unit, Critical care

## Abstract

**Background:**

POINCARE-2 trial aimed to assess the effectiveness of a strategy designed to tackle fluid overload through daily weighing and subsequent administration of treatments in critically ill patients. Even in highly standardized care settings, such as intensive care units, effectiveness of such a complex intervention depends on its actual efficacy but also on the extent of its implementation. Using a process evaluation, we aimed to provide understanding of the implementation, context, and mechanisms of change of POINCARE-2 strategy during the trial, to gain insight on its effectiveness and inform the decision regarding the dissemination of the intervention.

**Methods:**

We conducted a mixed-method process evaluation following the Medical Research Council guideline. Both quantitative data derived from the trial, and qualitative data from semi-structured interviews with professionals were used to explain implementation, mechanisms of change of the POINCARE-2 strategy, as well as contextual factors potentially influencing implementation of the strategy.

**Results:**

Score of actual exposure to the strategy ranged from 29.1 to 68.2% during the control period, and from 61.9 to 92.3% during the intervention period, suggesting both potential contamination and suboptimal fidelity to the strategy. Lack of appropriate weighing devices, lack of human resources dedicated to research, pre-trial rooted prescription habits, and anticipated knowledge of the strategy have been identified as the main barriers to optimal implementation of the strategy in the trial context.

**Conclusions:**

Both contamination and suboptimal fidelity to POINCARE-2 strategy raised concerns about a potential bias towards the null of intention-to-treat (ITT) analyses. However, optimal fidelity seemed reachable. Consequently, a clinical strategy should not be rejected solely on the basis of the negativity of ITT analyses’ results. Our findings showed that, even in highly standardized care conditions, the implementation of clinical strategies may be hindered by numerous contextual factors, which demonstrates the critical importance of assessing the viability of an intervention, prior to any evaluation of its effectiveness.

**Trial registration:**

Number NCT02765009

**Supplementary Information:**

The online version contains supplementary material available at 10.1186/s12874-024-02288-1.

## Background

Randomized controlled trials (RCT) provide clinical evidence on the effectiveness of new treatments and procedures in the most rigorous way [1]. However, many questions may arise from the results and are not addressed by the trial itself: why and how does the intervention work, or not? And if proved effective, can the intervention apply to general care situations [2]? These questions are especially relevant when considering complex interventions, whose various interconnecting components and interaction with context may interfere with the trial outcomes [3–8]. Through means of a process evaluation, it is possible to address these aspects by switching the focus from the “binary question of effectiveness” [9] to the implementation process and transferability of complex interventions [8], enabling stakeholders to make the most appropriate decisions regarding the dissemination of these interventions.

Stepped-wedge RCT POINCARE-2 aimed to assess the effectiveness of a strategy targeting fluid balance on 60-day mortality in patients requiring intensive care. [10] This strategy spanned over fourteen days, and consisted of daily weighing and subsequent decision of fluid input restriction and/or administration of diuretics, albumin, or fluid balance control during renal replacement therapy if any. [10] The intervention offered room for adaptation, as intensivists could readjust the strategy according to patient’s clinical condition and contraindications. Despite a lower 60-day mortality rate observed in the intervention group (30.5%, 95% confidence interval [CI], 26.2 to 34.8), we noticed no statistically significant difference as compared to the control group (33.9%, 95% CI, 29.6 to 38.2) in intention-to-treat (ITT) analyses [11].

ITT analyses allow firm conclusions about intervention effectiveness in case of significant results [12]. However, their validity may be threatened by the implementation of complex interventions, such as the POINCARE-2 strategy, involving multiple health professionals and multiple tasks. In fact, such interventions are known to be sensitive to context [6] and to usually depart from any standardization attempt [4], which might result in both suboptimal implementation and contamination [12]. Besides, these phenomena can be exacerbated by a stepped-wedge design of the trial. Such designs are useful when implementing strategies whose supposed effectiveness is based on consensus, such as fluid-balance control, to avoid non-adherence of centers that are at risk at the time of randomization of ending up in the control group and never benefiting from the strategy, but increase the risk of contamination in clusters awaiting implementation of the strategy [13]. Accordingly, on the ground that ITT analysis is sufficient per se to conclude about the actual relevance of a strategy in clinical practice [2], the risk is to discredit a strategy that might actually be effective. A thorough process evaluation of contextual factors, implementation (fidelity, dose, reach, adaptation), and mechanisms underpinning the effect of the of POINCARE-2 strategy on the outcomes could help understand: (i) the extent to which ITT analyses results might be biased towards the null; (ii) whether the strategy can actually be optimally implemented or not in daily Intensive Care Unit (ICU) practice; and (iii) contextual factors influencing its implementation to address eventual transferability issues in case of actual effectiveness. Process evaluations of interventions carried out in critical care settings are scarce, probably because care practices are a priori highly standardized and patients are mostly compliant. However, ICUs remain a complex environment, partly due to the complexity and severity of the pathologies treated, and it is crucial to investigate the factors that may hinder implementation of a complex clinical intervention in such settings.

Using a dedicated process evaluation as recommended by the Medical Research Council (MRC) [8], we aimed to assess contextual factors, implementation, and mechanisms of change of the POINCARE-2 strategy, assess the extent to which they impacted its effects on the trial outcomes, and question the conditions of implementation that might have allowed us to draw conclusions about the strategy potential effectiveness.

## Methods

### Setting: POINCARE-2 trial

The POINCARE-2 trial was a stepped-wedge randomized controlled trial carried out between June 2016 and May 2019, aiming to assess the effectiveness of a fluid balance control strategy performed in the post-acute phase (more than 48 h after admission) on 60-day mortality in mechanically ventilated patients. The trial protocol has been extensively described elsewhere. [10] Briefly, the strategy under scrutiny consisted of daily weighing over a fourteen-day period after admission to an ICU to assess fluid balance, and subsequent daily decision of (i) abstinence of specific intervention in case of fluid balance; or (ii) restriction of fluid input, administration of diuretics and albumin, and/or fluid balance control during renal replacement therapy if any, in case of fluid overload. We compared this strategy to standard of care, i.e., weekly weighing, and no particular protocol of care to handle potential fluid overload. We assessed the main outcome, i.e., 60-day mortality, using ITT analyses.

To draw the centers intent to participate in the trial, all investigators had access to the strategy algorithm as part of the protocol submitted to the DGOS-PHRC-N-2014 *(Direction Générale de l’Offre de Soins-Programme Hospitalier de Recherche Clinique-National)* call for funding prior to its implementation. A total of 1,361 patients in twelve French ICUs were included in the trial [11].

### Design

**Fig. 1:**
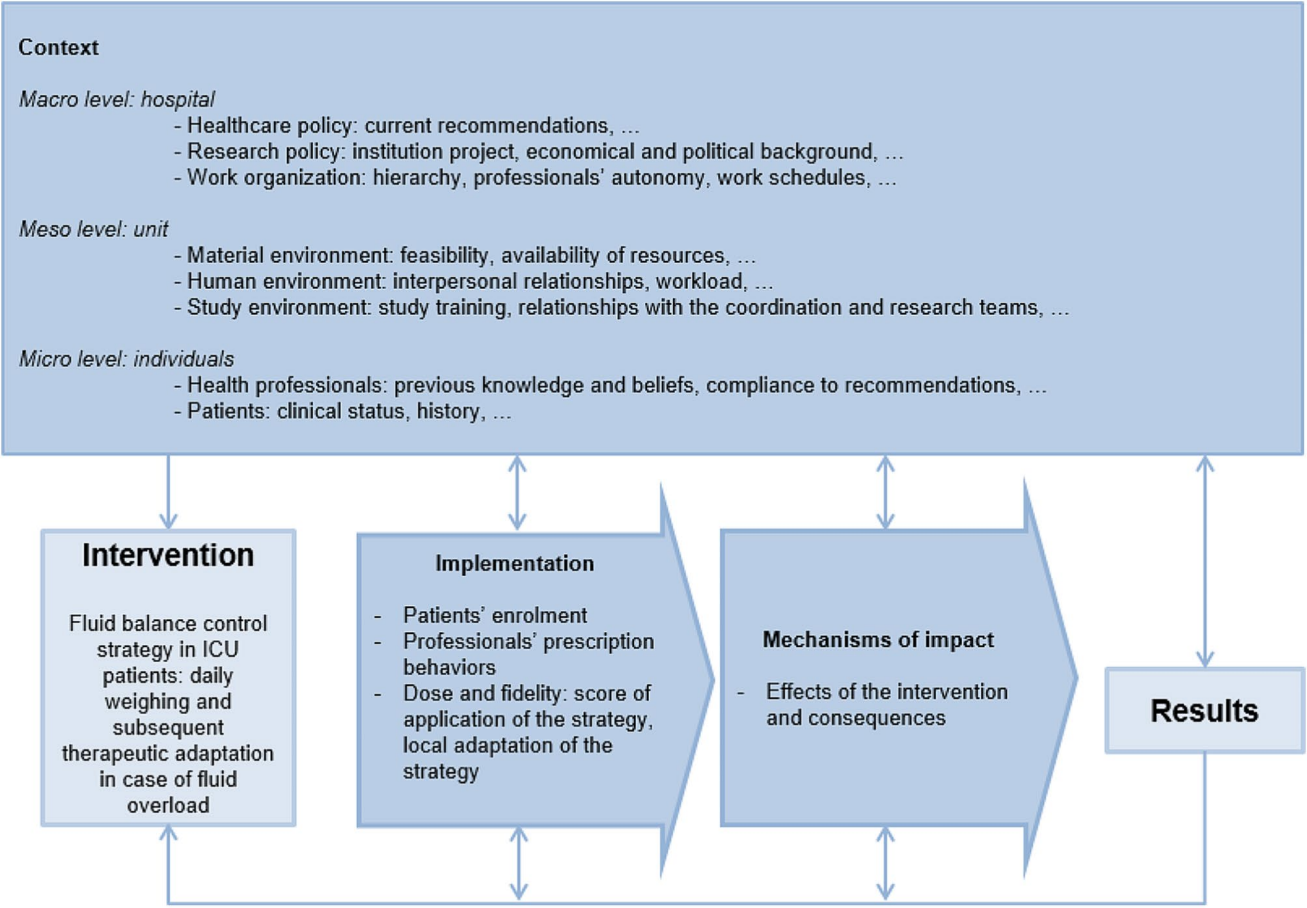
Schematic representation of the process evaluation framework for the POINCARE-2 trial, adapted from the Medical Research Council guidance [7]

Based on the Medical Research Council (MRC) model for process evaluations [5, 7, 8], and on May’s normalization process theory [14], an evaluation framework specific to the POINCARE-2 trial was designed (Fig. 1) to investigate context, implementation, and mechanisms of change of the strategy. Process evaluations are helpful to (i) explain how an intervention works, i.e. describe the different factors impacting its success or failure; (ii) assess the quality of implementation, i.e. subcomponents of fidelity, dose, adaptation, and reach; and (iii) determine whether the observed results are actually due to the intervention itself, or to implementation circumstances, i.e. context. [7] We used mixed methods, i.e., both quantitative data derived from the trial Case Report Forms (CRF), and qualitative data retrieved from semi-structured interviews with health professionals.

The Good Reporting of A Mixed Methods Study (GRAMMS) recommendations were used to report this process evaluation [15] (Supplement 1 in Appendix).

### Participants

We considered all patients from all participating ICUs for quantitative analysis.

While the aim of generalization in quantitative approaches requires statistical techniques to estimate minimum sample size, the goal of qualitative investigations is rather, according to Polit and Beck [16], to “provide a rich, contextualized understanding of some aspect of human experience”, and grasp the diversity of particular cases, to ensure transferability, i.e. extrapolation of findings to new situations or contexts. Consequently, qualitative research sample size estimation is based on the number of participants needed to achieve saturation of concept [17], defined as the point where no new information emerges in the data analysis process, and further data collection would yield similar results and may be redundant. [18] A comprehensive review showed that most qualitative studies reached saturation under thirty interviews. [19] We therefore decided to perform interviews with health professionals involved in the trial implementation, i.e., local investigators (intensive care physicians responsible for the conduct of the trial in their ICU), ICU head nurses, physicians specialized in intensive care medicine, nurses, assistant nurses, and medical assistants, in a minimum of four ICUs (33% of the ICUs) and up to saturation. ICUs for qualitative interviews were selected for the variety of their characteristics and recruitment rate during the trial, following a maximum variation sampling approach, in order to “identify important common patterns that cut across variations” [20–22]. We undertook initial analyses simultaneously with data collection to ensure data saturation had been achieved.

### Data collection

#### Quantitative outcomes

We used quantitative data to assess implementation of the strategy and its mechanisms of change (Fig. 1).

The number of expected and included patients during each period (control vs. strategy) were used to assess strategy reach.

Length of ICU stay, completion of daily weighing, occurrence of arterial hypotension, daily levels of serum sodium and potassium, level of the RIFLE classification, dose of diuretics, oral or parenteral intake volume (except for vascular filling), and ultrafiltration volume were used to calculate a score of actual exposure to the strategy to assess fidelity and dose during the intervention period, and contamination during the control period. Fidelity to the strategy was also assessed by the number of weekly weighing per patient and the proportion of weighing performed (i.e., achieved over expected).

We assessed mechanisms of change using patient weight change between admission and discharge, and proportion of patients who presented at least one episode of arterial hypotension during their ICU stay, the latter being considered both as the main deleterious side effect and the main contra-indication to the intervention.

### Qualitative data

MB and LR carried out semi-structured interviews from March to July 2020. Investigators from the four selected ICUs were invited to participate and were asked to invite at least one professional for each profession of interest from their ICU to participate. Based on our framework (Fig. 1), we developed interview guides with variations depending on the profession of participants.

Both interviewees and interviewers were blinded to the results of the trial at the time of the interviews, and both researchers who conducted the interviews had no relationship with the interviewees before the process evaluation.

### Data analyses

We summarized quantitative data with descriptive statistics using SAS 9.4 software (SAS Institute, Inc., Cary, NC).

We considered that exposure to the strategy was consistent with the protocol if a minimum 30% restriction of water and salt intake as compared to the previous day, or prescription of diuretics, or ultrafiltration, was applied on any given day between Day 2 and Day 14 after admission, when patient’s daily weight exceeded a predefined threshold (admission weight + 2 kg on Day 2, admission weight from Day 3 to Day 7, or admission weight – 2 kg from Day 8 to Day 14). For each day spent in the ICU over this 12-days period, and for each included patient, we accounted for every observed deviation to the strategy, defined as non-application of the above intervention when it should. In case of contraindication to water and salt restriction (arterial hypotension, serum sodium > 155 mmol/L, serum potassium < 2.8 mmol/L, or onset of renal dysfunction ≥ « risk » level of the RIFLE classification), no deviation was accounted for. To account for various lengths of stay, we divided this first score by the number of days spent in the ICU and transformed it to range from 0 to 100. Finally, it was weighted by the proportion of daily weigh-ins performed between Day 2 and Day 14, leading to the final score of actual exposure to the strategy, ranging from 0% (minimal exposure, i.e., minimal dose of strategy delivered) to 100% (maximal exposure, i.e., maximal dose of strategy delivered). To assess both usual practices regarding fluid balance control before the trial, and fidelity to the strategy protocol, we computed a mean score for each ICU during both the control and intervention periods, respectively.

Interviews were audio recorded and transcribed verbatim, anonymously. Based on our framework (Fig. 1), MB and LR conducted a thematic analysis using NVivo^®^ V11. They developed a coding tree using a combination of deductive and inductive approaches [23], and achieved the coding process independently for five interviews, enabling the coding tree to be progressively refined, based on discussion and solving of disagreements. We calculated Cohen’s Kappa to measure intercoder agreement, with a desired value > 0.81 [24]. MB then coded the remaining transcripts.

We performed quantitative and qualitative analyses simultaneously and triangulated them, following a concurrent design [25].

### Ethical considerations

All health professionals gave oral consent to participate in the qualitative approach.

## Results

MB and LR conducted twenty interviews in four ICUs with four investigators, three physicians, three head nurses, four nurses, four assistant nurses, and two medical assistants (Table 1). Intercoder agreement was very satisfactory with a Cohen’s Kappa at 0.97.

Illustrative quotes are gathered in Table 2. For each identified factor, we determined whether it was a facilitator or, on the opposite, a barrier to the implementation of the strategy (Table 2). Strategy implementation and mechanisms of change are described in Table 3. For Tables 1, 2 and 3, we randomly assigned ICUs with a letter between A and L regardless of their randomization order in the trial, to ensure anonymity.

**Table 1:** Descriptive characteristics of the qualitative sample used in the POINCARE-2 process evaluation

ICU	*N*	Profession	Abbreviation
ICU D	4	1 investigator 1 physician 1 nurse 1 assistant nurse	INV-13
			MED-8
			NU-4
			ANU-2
ICU B	4	1 investigator 1 head nurse 1 nurse 1 assistant nurse	INV-16
			HNU-20
			NU-18
			ANU-12
ICU C	5	1 investigator 1 physician 1 nurse 1 assistant nurse	INV-17
			MED-7
			NU-6
			ANU-5
		1 head nurse	HNU-10
ICU H	7	1 investigator 1 physician 1 nurse 1 assistant nurse	INV-3
			MED-1
			NU-9
			ANU-11
		1 head nurse	HNU-14
		2 medical assistants	MAS- 15 & MAS-19
Total	20	4 investigators 3 physicians 4 nurses 4 assistant nurses 3 head nurses	
		2 medical assistants	

**Table 2:** Factors pertaining to the context, implementation, and mechanisms of change of the POINCARE-2 strategy: illustrative quotes, and perceived impact on the trial application

Theme	Factor	Perceived Impact	Quotes
Macro contextual factors	Research policy	Facilitator in teaching hospitals only	**Center C, INV-17**: “That’s why we’re very keen to do research, to attract young practitioners… they must have research to do if we want them to stay.” “It would be interesting for us to have more of a research policy with other units [of the same hospital complex], because there are practitioners who are experienced in research, they could give us the benefit of their experience…”
	Work organization	Barrier in non-teaching hospitals	**Center C, MED-7**: “Since we don’t have enough practitioners in our unit, we are pretty limited to do more research […] our priority is to fill in our shifts schedule.”
Meso contextual factors	Units layout	Facilitator	**Center C, MED-7**: “[The break room] is very convenient, you get to share important information with the whole team at the same time.”
	Strategy algorithm display	Facilitator	**Center D, INV-13**: “The nurses couldn’t not be aware… The posters were even stuck on the walls, so they remained for a while after the end of the trial…”.
	Equipment quality and availability	Barrier	**Center B, NU-18**: “Clearly POINCARE-2 did not go very well with the caregivers […] because of the daily weighing, we had to wait until the lifter was available, and we didn’t have enough straps to weigh either… So, it was hell, every morning… it wasn’t so much the weighing that was the problem, but the lack of equipment, and the fact that we had to wait on top of each other…. “ **Center B, INV-16**: “The problem of weighing in ICU is endless.”
	Hierarchical patterns and inter-professional relationships	Facilitator	**Center C, HNU-10**: “It’s something that is quite obvious in ICU. There is a real partnership between all care providers, medical and paramedical staff.”
		Barrier	**Center B, HNU-20**: “Here in the department, we have 3 lifters, each of which is allocated to a unit, so when you have to weigh all patients every day, there are 25 patients to weigh, so you set up the first one, the patient lifter is on the other side, so you have to wait, etc…. then there’s the equipment decontamination time, which isn’t negligible, because otherwise you risk transmitting diseases to others, so it was quite difficult, and as a result, that put a bit of tension within the team… not so much within the paramedical team, but between the paramedical team versus the medical team.”
	Workload	Barrier for nurses and assistant nurses	**Center B, ANU-12**: “It’s true that it was a lot of work, but we’re not in the ICU to have fun.”
			**Center D, MED-8**: “We didn’t hear it, well, I didn’t get any feedback from the nurses on the workload related to the protocol.”
	Turnover	Barrier	**Center B, HNU-20**: “They [young health professionals] have their head under water the first year, so you might as well say that clinical research is really not their priority. […] The team’s youth is really an obstacle to research, even though in reality they have significant added value.”
	Opinion leader(s)’ behavior	Facilitator	**Center D, INV-13: “**Everything is noted in the rooms, and I make sure that the protocols are applied when I am responsible for them.” **Center H, INV-3**: “I just asked my colleagues to do the inclusions. And I took care of everything else.”
	Scientific dynamic	Facilitator in teaching hospitals	**Center B, NU-18**: “Sometimes we have up to 20 different trials at the same time in our unit, so we’re accustomed to it.”
	ICU usual practices regarding fluid balance	Barrier when no adaptation	**Center C, MED-7**: “We know fluid balance is part of the prognosis for resuscitation patients.” “Albumin use is uneven in our unit since it was not proven to be systematically effective, but it seems to help.”
	Study training	Facilitator for medical staff	**Center C, INV-17**: “Coordination team was really helpful.”
	Attitudes towards research teams	Barrier for paramedical staff (lack of communication) Facilitator for investigators	**Center B, HNU-20**: “It’s true that, what is really missing is communication about research, its benefits, and results.” **Center H, INV-3**: “I had excellent relations with the research team; they would answer every time I called.”
Micro contextual factors	Clinical experience	Facilitator	*Supplementary Material #5*
	Research experience	Facilitator in teaching hospitals only	**Center C, MED-7**: “I’m more of a clinical physician, so I’m very involved in the department, but organizing a trial is not something I’d do. […] But on the other hand, I am always interested in following a protocol; if I’m offered one, I try to do it.”
	Professional’s dedication and motivation	Facilitator when positive	**Center B, INV-16**: “For us physicians, it would be really helpful if patients were weighed every day, but there is the technical problem for the paramedical staff, with the induced [additional] workload” **Center H, HNU-14**: “The team quickly realized that such a study was interesting, and although it would probably be very time-consuming, it was part of the game in an ICU.” **Center C, ANU-5**: “I would say it’s mixed… we were conscientious to do it properly, but whether we felt really involved or not… really a mixed feeling.” **Center B, HNU-20**: “Adherence [by nurses and assistant nurses] to the project is sometimes a little bit difficult, because it is not always well explained…”
	Professional’s knowledge and attitudes towards fluid balance control	Facilitator when positive	**Center C, MED-7**: “Studies with albumin, [they showed] no benefit of doing it systematically.” **Center C, INV-17**: “I am very much involved in the weighing of patients, and especially input-output assessments, in my case it is systematic. […] weight is one element but it’s not everything.”
	Professional’s attitudes towards institution and unit policies	Barrier in non-teaching hospitals	**Center C, INV-17**: I think someone from hospital management asked me what POINCARE-2 was, and I honestly didn’t answer… they don’t give a damn anyway.
	Professional’s attitudes towards the trial execution	Facilitator when positive	**Center D, MED-8**: “One can criticize the study, but I think it had the courage to try to prove something that nobody had yet managed to prove.” **Center H, MED-1**: “We’d never do it again because, honestly, it’s too much work.”
	Patient-related factors (as perceived by health professional)	Barrier	**Center B, ANU-12**: “Weighing can be painful […] sometimes it is also inconvenient for the patient, because it hurts him” **Center B, NU-18**: “It’s dangerous […] it’s always risky to weigh a patient.” **Center C, ANU-5**: “Well, if we can’t stabilize the patient [in terms of arterial pressure] … we’re not going to allow ourselves to lateralize him on one side, turn him on the other side, put him on the lifter… at the end of all that, the patient wouldn’t tolerate it.” **Center H, NU-9**: “With our devices, we organize ourselves quickly now … we get together with several nurses if the patients are unstable … we all go in and then we weigh them quickly.” Center D, NU-4: “[Before the trial] we weighed patients twice a week, except for dialysis patients, whom we knew we needed to monitor specifically, we weighed them every day.”
Implementation	Patients enrolment		**Center C, MED-7**: “We had some problems with enrolment, especially when there was too much work… At one point we were late, and the coordination team told us so, and I remember telling them I couldn’t do better since we had not enough eligible patients.”
	Protocol application		**Center B, INV-16**: “We did everything we could to achieve weight loss, but perhaps not by following the algorithm because it did not match our weight loss strategies.”
	Evolution of resources		**Center B, HNU-20**: “The trial actually helped us since we could tell management that we required more resources for research. But it only changed after the trial.”
Mechanisms of change	Effects of the intervention and consequences		**Center H, INV-3**: “We used to bring way too much salt to patients, so the trial definitely helped us with that.”

**Table 3:** Quantitative outcomes about POINCARE-2 strategy reach, dose and fidelity, and mechanisms of change in each participating ICU

ICU	Reach			Dose and fidelity							Mechanisms of change				
	Patients included/ expected (%)		Average number of weekly weigh-ins per patient Mean (SD)			Weighing Achieved /Expected (%)		Score of actual exposure to the strategy (range 0-100%) Mean (SD)		Proportion of patients with at least one episode of hypotension during ICU stay % [*N*]			Weight change between ICU admission and discharge (kg) Mean (SD)		
	Control	Intervention	Control		Intervention	Control	Intervention	Control	Intervention	Control		Intervention	Control	Intervention	
**A**	143.3	78.3	4.9 (1.3)		5.2 (1.2)	442.6	87.7	68.2 (19.6)	73.7 (20.2)	97.7 [84]		97.9 [46]	-0.8 (6.8)	-0.1 (7.4)	
**B**	151.7	143.3	4.1 (1.5)		5.8 (1.2)	373.8	97.3	61.2 (20.4)	87.4 (15.5)	95.6 [87]		94.2 [81]	-1.9 (8.0)	-2.1 (6.6)	
**C**	86.7	81.7	2.2 (1.3)		5.4 (1.2)	193.3	89.0	29.1 (20.5)	73.4 (19.4)	92.3 [48]		100.0 [49]	-4.9 (9.0)	-1.6 (7.8)	
**D**	58.3	48.3	4.1 (1.4)		5.2 (1.5)	387.6	77.0	61.7 (24.0)	61.9 (19.1)	94.3 [33]		72.4 [21]	0.2 (7.7)	0.0 (6.7)	
**E**	73.3	98.3	2.7 (1.2)		5.9 (1.2)	240.2	91.6	37.9 (18.8)	85.8 (13.3)	72.7 [32]		76.3 [45]	-2.8 (5.3)	-3.6 (3.5)	
**F**	173.3	96.7	3.6 (1.1)		4.7 (1.4)	309.0	80.0	44.5 (16.4)	63.7 (20.9)	93.3 [97]		96.6 [56]	1.3 (9.0)	-1.0 (9.6)	
**G**	73.3	81.7	5.1 (1.6)		5.6 (1.1)	439.4	93.5	61.5 (24.1)	78.5 (19.4)	88.6 [39]		83.7 [41]	-1.3 (8.8)	-2.9 (6.0)	
**H**	58.3	41.7	2.2 (1.0)		5.9 (1.2)	190.5	95.9	31.2 (16.9)	92.3 (14.2)	82.9 [29]		68.0 [17]	-3.6 (7.3)	-4.6 (8.8)	
**I**	101.7	95.0	3.4 (1.5)		5.4 (1.4)	313.7	90.2	49.9 (22.5)	77.5 (19.5)	80.3 [49]		82.5 [47]	-1.6 (5.6)	-2.7 (6.6)	
**J**	118.3	118.3	4.8 (1.4)		5.5 (1.2)	418.8	89.2	65.9 (19.8)	78.5 (19.4)	91.5 [65]		98.6 [70]	-0.2 (8.7)	-1.5 (8.7)	
**K**	81.7	108.3	4.2 (1.1)		5.1 (1.2)	359.5	84.0	56.5 (14.3)	73.4 (18.0)	98.0 [48]		98.5 [64]	-0.3 (9.6)	-1.0 (8.2)	
**L**	76.7	80.0	2.4 (0.9)		4.8 (1.5)	270.3	83.8	39.2 (15.2)	73.6 (22.7)	82.6 [38]		85.4 [41]	1.5 (8.5)	-0.7 (7.4)	

### Context

Main contextual factors that might have influenced strategy implementation or mechanisms of change (Fig. 1) are detailed below and summarized in Table 4.

**Table 4:** Major contextual factors influencing the implementation of the POINCARE-2 strategy

Level	Contextual Factor	Impacted implementation process	Nature of impact
Macro (Hospital)	Teaching hospital (or not)	Data collection Strategy application	Contamination (or suboptimal application)
	Resources dedicated to research	Data collection Strategy application	Compliance with protocol
Meso (ICU)	Units layouts	Strategy application	Suboptimal application
	Equipment quality and availability	Strategy application	Contamination (availability of scale-beds) Suboptimal application (lack of weighing scales)
	Inter-professional relationships	Strategy application	Compliance with the protocol
	Usual practices regarding fluid balance control	Strategy application	Contamination (embedded in usual practices) Suboptimal application (not part of usual practices)
	Anticipated knowledge of the strategy	Strategy application	Contamination
	Staff turnover	Strategy application	Suboptimal application
	Workload	Strategy application Data collection	Compliance with protocol
Micro (Individuals)	Professional’s dedication and motivation	Patient recruitment Strategy application	Compliance with protocol
	Professional’s knowledge and attitudes towards fluid balance control	Strategy application	Contamination (expertise/positive attitudes) Suboptimal application (insufficient knowledge/negative attitudes)
	Patient’s clinical conditions	Strategy application	Contamination (indication to daily weighing) Suboptimal application (perceived or actual contra-indication to daily weighing)

### Macro contextual factors: hospital

Implementation of the strategy seemed eased in teaching hospitals, which benefited from larger medical teams, dedicated research staff, and support from their institution. Implementation of the trial in non-teaching hospitals required adaptations, mainly because of the lack of human resources dedicated to data collection, unraveling medical resources towards non-medical tasks pertaining to the protocol, which may have, in the end, hindered patients’ recruitment.

*“Since we don’t have enough practitioners in our unit, we are pretty limited to do more research […] our priority is to fill in our shifts schedule.”* (Center C [non-teaching hospital], MED-7) (Table 2).

### Meso contextual factors: Intensive Care Unit

#### Physical environment

ICU layout, strategy algorithm displays, and equipment quality and availability were reported as critical factors that might have influenced implementation of the strategy and are detailed in Supplement 2 in Appendix.

For instance, the investigated strategy relying on daily weighing, quality and availability of weighing equipment were critical to its execution. However, professionals reported a lack of adequate equipment in all visited ICUs, which led to missed weigh-ins, measurement bias – because of electronic malfunctions or problems with tare – and an increased workload for nursing staff, resulting in a suboptimal application of the strategy, as evidenced by the weekly average number of weigh-ins during the intervention period which departed from seven in all ICUs (Table 3). Inadequate communication between nursing and medical staff might have maintained this equipment-related hurdle (Supplement 3 in Appendix).

*“[…] Because of the daily weighing, we had to wait until the lifter was available, and we didn’t have enough straps to weigh either… So, it was hell, every morning… it wasn’t so much the weighing that was the problem, but the lack of equipment, and the fact that we had to wait on top of each other…”* (Center B, NU-18) (Table 2).

### Human environment

Hierarchical patterns and inter-professional relationships, workload, staff turnover, opinion leader’s attitudes towards the strategy, and previous ICU practices towards fluid balance that might also have influenced implementation of the strategy are detailed in Supplement 3 in Appendix.

Nurses’ feeling to be ignored by physicians when reporting problems with daily weighing might have led to inter-professional relationship pitfalls hindering fidelity to the strategy.

*“Here in the department, we have 3 lifters, each of which is allocated to a unit, so when you have to weigh all patients every day, there are 25 patients to weigh, so you set up the first one, the patient lifter is on the other side, so you have to wait, etc…. then there’s the equipment decontamination time, which isn’t negligible, because otherwise you risk transmitting diseases to others, so it was quite difficult, and as a result, that put a bit of tension within the team… not so much within the paramedical team, but between the paramedical team versus the medical team.”* (Center B, HNU-20) (Table 2).

Staff turnover, especially bi-annual turnover of residents, also curbed the strategy implementation.

*“[Residents] have their head under water the first year, so you might as well say that clinical research is really not their priority. […] The team’s youth is really an obstacle to research, even though in reality they have significant added value.”* (Center B, HNU-20) (Table 2).

Standard of care regarding fluid balance varied considerably from one ICU to another, as shown by the weekly average number of weigh-ins and the score of actual exposure to the strategy during the control period (Table 3), leading to some contamination in most ICUs.

### Study environment

Scientific dynamic, anticipated knowledge of the strategy because of the stepped-wedge design, satisfaction with training to the strategy, and differential attitudes towards the research team between nursing and medical staff, might have affected implementation of the strategy and are detailed in Suppl.3.

In two ICUs, all physicians knew about the strategy before the trial began, because of the steeped-wedge design, and had started to change their practices regarding fluid balance control during the control period, which may have led to contamination.

### Micro contextual factors: individuals

#### Health professionals

Professionals’ attitudes towards hospital and ICU policies, their clinical and research training and experience, their dedication and motivation towards research, and their knowledge and attitudes towards fluid balance, might have curbed the implementation of the strategy and are detailed in Supplement 5 in Appendix.

Tasks of the strategy involving nurses and assistant nurses, i.e., daily weighing, were, as a first step, critical to the strategy implementation. However, nurses and assistant nurses undervalued their own role in the trial.

Moreover, some professionals were reluctant to apply the strategy as planned because it did not conform to their own therapeutic habits. Others were used to apply water and salt restriction and might have applied the strategy long before the intervention period had started, causing another source of contamination (Table 3).

*“I am very much involved in the weighing of patients, and especially input-output assessments, in my case it is systematic. […] weight is one element but it’s not everything.”* (Center C, INV-17) (Table 2).

### Patients

Patients’ health status could have impacted the strategy implementation, both by preventing daily weighing of most vulnerable patients when weighing was performed with a lifter, for reasons of safety; but also, by inducing contamination, for instance for patients with end-stage renal disease who were already weighed daily before the trial in all ICUs.

*“Well, if we can’t stabilize the patient [in terms of arterial pressure] … we’re not going to allow ourselves to lateralize him on one side, turn him on the other side, put him on the lifter… at the end of all that, the patient wouldn’t tolerate it.”* (Center C, ANU-5) (Table 2).

*“[Before the trial] we weighed patients twice a week, except for dialysis patients, whom we knew we needed to monitor specifically, we weighed them every day.”* (Center D, NU-4) (Table 2).

### Implementation

#### Reach: patients’ enrolment

Inclusion varied, as evidenced by the proportion of recruited patients among expected enrolment (Table 3). One ICU had to relocate during the intervention phase, which hindered its inclusion rate. Two physicians conceded that recruitment was more difficult during periods with work overload, but also during weekends, as on-call physicians did not always apply the strategy.

*“We had some problems with enrolment, especially when there was too much work… At one point we were late, and the coordination team told us so, and I remember telling them I couldn’t do better since we had not enough eligible patients.”* (Center C, MED-7) (Table 2).

None of the interviewed physicians reported any reluctance to include a patient related to the strategy under scrutiny during the intervention period.

#### Fidelity to the strategy and dose

Fidelity to the strategy is described in Table 3. Mean score of actual exposure to the strategy was higher during the intervention period than during the control period in all ICUs. Mean score varied from 29.1 to 68.2% (control) vs. from 61.9 to 92.3% (intervention), highlighting (i) contamination during the control period, (ii) suboptimal application of the strategy during the intervention period, and (iii) feasibility of quasi-optimal implementation in daily practice. However, for ICUs that scored over 50% during the control period, potential of improvement was limited. One investigator agreed that the strategy may not have been fully executed during the intervention period, mostly because the algorithm did not correspond to the physicians’ prescriptions habits, leading to reduced adherence to the protocol.

*“We did everything we could to achieve weight loss, but perhaps not by following the algorithm because it did not match our weight loss strategies.”* (Center B, INV-16) (Table 2).

For instance, several of them were reluctant to prescribe diuretics when natremia exceeded 150 mmol/L, despite the protocol mentioning they could be used up to 155 mmol/L. Of note, although the use of diuretics is associated with an increased risk of hypernatremia [26], and hypernatremia is known to be an independent risk factor for mortality [27], there is to date no clear recommendation in the literature regarding the threshold of natremia beyond which the use of diuretics should be limited. The near-optimal score obtained in ICU C (92.3%) could be explained by the significant commitment made by a local physician, who ensured strict compliance with the protocol by all professionals.

“So I took care of the follow-up […] we realized very quickly that someone had to be in charge, otherwise we would have been excluded from the study. My colleagues, I just asked them to include patients. And I took care of everything.” (Center H, MED-1).

#### Adaptation

In one ICU, the trial was used to claim additional weighing beds, that came in after the end of the trial (Table 2). In one ICU from a non-teaching hospital, the investigator had reworded the algorithm underpinning the strategy before display to facilitate its appropriation by physicians, nurses, and assistant nurses (Supplement 2 in Appendix).

### Mechanisms of change: unintended and intended intermediate effects of the strategy

Relying on fluid restriction, POINCARE-2 strategy could theoretically cause hypotension. However, four ICUs experienced a reduction in the incidence of hypotension during the intervention period, including one center with an almost optimal score of actual exposure to the strategy (Center H), probably due to an increased attention paid to fluid balance (Table 3). In the other ICUs, a moderate increase in the incidence of hypotension was observed, which might explain suboptimal application of the strategy during the intervention period in these centers (C and K in Table 3).

In ten ICUs, as expected when particular attention is paid to fluid balance control, mean weight loss during ICU stay was higher in the intervention period as compared to the control period (Table 3). Accordingly, despite suboptimal application, the strategy seemed to have had a perceptible effect on intermediate outcomes.

Three physicians indicated that their attitudes towards about fluid balance had changed thanks to the trial.

*“We used to bring way too much salt to patients, so the trial definitely helped us with that.”* (Center H, INV-3) (Table 2).

In one ICU, the trial reinforced systematic daily weighing and the perceived value of albumin. Other physicians, as well as nurses and assistant nurses, considered that the trial did not fundamentally change their habits since they already paid careful attention to fluid balance before. As a result, professionals from most ICUs reported that the frequency of weighing had returned to the previous rate after the end of the study.

## Discussion

This process evaluation offered a better understanding of contextual factors, implementation, and mechanisms of change influencing the effect of the POINCARE-2 strategy on the trial outcomes. Its findings shed new light on the negative results of ITT analyses [11], highlighting both contamination during the control period and suboptimal application of the strategy during the intervention period in all ICUs. This is likely to explain the extent to which ITT analyses biased the main results towards the null and underestimated the effect of the intervention, by considering patients from the control period unexposed to the strategy, and patients from the intervention period exposed to the strategy as planned, despite a mean score of actual exposure departing from its optimal value.

In one ICU, mean score of actual exposure to the strategy was almost maximal (92.3%), which argues in favor of the feasibility of optimal implementation of such a strategy in daily practice, at least in favorable contexts. Besides, mechanisms of change, such as mean weight loss and incidence of arterial hypotension, tended to credit POINCARE-2 strategy. Taken together, these findings further argue in favor of new analyses before discarding the strategy based on ITT analyses only, and highlight the need of a better understanding of contextual factors to assess whether the strategy could be fairly implemented in any ICU.

This process evaluation, through its qualitative approach, gave insights about such contextual factors that might explain both the observed contamination during the control period and the suboptimal application during the intervention period. Some of these factors, such as professionals’ attitudes towards fluid balance control and equipment availability or quality, might be used as levers to improve strategy implementation, or to refine the intervention so that it is better adapted to local constraints. Importantly, our results emphasize the fact that interventions cannot be implemented without first evaluating their viability, in terms of available material resources (for example, sufficient number of weighing beds), favorable inter-professional organization (for example, setting up procedures to improve feedback from nurses to doctors), and favorable work attitudes (for example, clarifying the implications of the intervention for patients to involved professionals). This invisible preparatory time, which is often not described as a component of the assessed interventions, is nonetheless indispensable, in addition to being time-consuming and costly. Evaluations are all too often concerned with what did not work, but the challenge lies beforehand: how can we ensure that the intervention is viable before it is effective?

### Strengths

One of the strengths of our work lies in its originality in terms of implementation: the context of intensive care. To our knowledge, this study is only the second one in the field of intensive care medicine to use a process evaluation to assess a complex intervention in a RCT so far. [28] This scarcity may reflect a lack of awareness of the adaptability of complex interventions, in a context accustomed to the application of standardized protocols and care.

Contrary to what we might initially have thought, even in a setting where standardized protocols are common practice, when the intervention has been developed by the professionals themselves, when it offered room for adaptation to match frequently encountered clinical care situations, and when the professionals have read the trial protocol before taking part (and are a priori convinced of the intervention’s benefits), we still observe problems of adherence and organization, resulting in insufficient implementation. Ultimately, this compromises the evaluation of the intervention’s effectiveness, making it impossible to determine whether the intervention is really ineffective, or whether the lack of effectiveness is in fact the result of a lack of power linked to a lack of implementation.

As recommended by the MRC [2, 5, 7, 8], we planned our process evaluation as part of the study protocol, and used mixed methods to widen the possibilities to capture any factor influencing the effect of the strategy on the outcomes. [29] We paid particular attention to the identification of mechanisms of change and resources required for the intervention, as well as the complexity arising from the many interactions between the intervention and its context of implementation.

Investigators were from different disciplines, addressing the need of interdisciplinary approaches in interventional research [30], and were blinded to the results of the main analysis while conducting the process evaluation, allowing complete independence.

### Limitations

Process evaluations should usually be conducted concomitantly to the trial to enhance monitoring of fidelity to the intervention. [31] However, due to lack of human resources, our investigations started after the trial. A process evaluation nested within the trial, with a formative rather than summative aim, could have enabled us to monitor protocol compliance more closely, and to propose adjustments or appropriate corrections when needed.

## Conclusions

As stated by Wells [6], “intervention description is not enough”, and understanding the context in which a complex intervention took place is crucial to the interpretation of the trial outcomes assessing its effectiveness. Our process evaluation of an intervention conducted in critical care settings showed that, even in highly standardized care conditions, the implementation of clinical strategies may be hindered by lack of resources, lack of adherence from professionals, and organizational shortcomings. Our findings also demonstrate the critical importance of assessing the viability of an intervention, prior to any evaluation of its effectiveness.

### Electronic supplementary material

Below is the link to the electronic supplementary material.

Supplementary Material 1

## Data Availability

No datasets were generated or analysed during the current study.

## Abbreviations

ICU: Intensive Care Unit
ITT: Intention-to-treat
MRC: Medical Research Council
RCT: Randomized Controlled Trial
