# Supplementary material for: Implementation of a fluid balance control strategy in critically ill patients: POINCARE-2 trial process evaluation
Source: BMC Med Res Methodol. 2024 Jul 24;24:160. doi: 10.1186/s12874-024-02288-1 (PMC11267841; doi:10.1186/s12874-024-02288-1)
Supplement: Supplementary file 1 — Supplementary Material 1 [file 12874_2024_2288_MOESM1_ESM.pdf]

SUPPLEMENTARY APPENDIX

TABLE OF CONTENTS

Supplement 1. Good Reporting of A Mixed Methods Study (GRAMMS) guideline 2

Supplement 2. Results about physical environment as a meso-contextual factor influencing POINCARE-2 strategy implementation 3

Supplement 3. Results about human environment as a meso-contextual factor influencing POINCARE-2 strategy implementation 5

Supplement 4. Results about study environment as a meso-contextual factor influencing POINCARE-2 strategy implementation 7

Supplement 5. Results about health professionals as a micro-contextual factor influencing POINCARE-2 strategy implementation 8

Supplement 1. Good Reporting of A Mixed Methods Study (GRAMMS) guideline

(1) Describe the justification for using a mixed methods approach to the research question	See Methods section, "Design" and "Participants" subsections (p. 7-9)
(2) Describe the design in terms of the purpose, priority and sequence of methods	See Methods section, "Data collection" subsection (p. 9-10)
(3) Describe each method in terms of sampling, data collection and analysis	See Methods section, "Participants" "Data collection" and "Data analyses" subsections (p. 7-11)
(4) Describe where integration has occurred, how it has occurred and who has participated in it	See Methods section, "Data analyses" subsection (p. 10-11)
(5) Describe any limitation of one method associated with the present of the other method	See Discussion section (p. 17-20)
(6) Describe any insights gained from mixing or integrating methods	See Discussion section (p. 17-20)

Supplement 2. Results about physical environment as a meso-contextual factor influencing POINCARE-2 strategy implementation

ICU layout

Units size varied from 8 to 25 beds. The available space between rooms was important to consider since all surveyed ICUs used patient lifters as weighing systems. These devices were usually stored and reloaded in corridors and needed to be transported quickly from one room to another to be used during patients' washing. Corridors width seemed sufficient in all ICU except for one, where traffic was difficult when several people crossed, which may have hindered optimal equipment use.

Strategy algorithm display

Strategy algorithm display and appropriation were good indicators of ICUs' commitment in the trial. The algorithm was displayed in every room in all visited ICUs. The investigator of one non-teaching hospital, whose team was not accustomed to research protocols, had reworded the algorithm to facilitate its appropriation by physicians, nurses, and assistant nurses.

Equipment quality and availability

The investigated strategy relying on daily weighing, quality and availability of weighing equipment were critical to the strategy execution.

Two types of weighing equipment were used: integrated-scale beds and patient lifters. All nurses and assistant nurses agreed that integrated-scale beds were much easier to use since they required less handling and no disinfection time, while patient lifters involved at least two caregivers. However, tare weight with integrated-scale beds greatly varied between professionals, and thus could bias weighing. Electronic malfunctions of weighing equipment were also reported in the four investigated ICUs, causing another source of bias.

The number of devices varied from one for three to one for eight patients and was lower in teaching hospitals. This lack of equipment was mentioned in all ICUs and led to a negative experience for non-medical staff. As a consequence, although the protocol stated that all weigh-ins should be performed with the same device for each patient, this could not always be done, since most of weigh-ins were performed simultaneously in the morning during patients' washings. Local adaptations were sometimes implemented to reduce the number of missed weigh-ins, by weighing patients at different times of the day. In two ICUs, covers or straps needed to use patient lifters were missing, which also prevented daily weighing.

These equipment difficulties were mostly experienced by nurses and assistant nurses, and then reported to medical staff and head nurses. However, half of the interviewed physicians did not perceive any equipment-related hurdle, although nurses from the same units did mention them, which may reflect different perceptions and a lack of communication between professionals.

Supplement 3. Results about human environment as a meso-contextual factor influencing POINCARE-2 strategy implementation

Hierarchical patterns and inter-professional relationships

Hierarchical patterns seemed harmonious in all visited ICUs. However, divergences emerged during interviews as to how professionals perceived the trial according to their status. Two physicians mentioned the nurses’ reluctance to deal with daily weighing because of the lack of equipment. One head nurse explained that this pitfall, on top of nurses' feeling about their complaint being ignored, stressed out the difficulties regarding relationships between nurses and physicians, that were likely to impact even further implementation of the strategy. Inter-professional relationships appeared more harmonious in non-teaching hospitals likely due to smaller staff. Nurses could therefore more easily express their difficulties to physicians who in return might be more receptive.

Communication between medical and nursing staffs mostly happened during daily medical visits. Head nurses were also a strong connection especially regarding communication about the trial and equipment management.

Workload

Workload was described by all professionals as a major obstacle to the trial implementation, especially nurses and assistant nurses, because of the additional work induced by daily weighing during the intervention period.

For medical staff, additional work was required for patients screening and data collection, particularly in ICUs where no dedicated research team was available. One physician conceded that quality of data collection depended much on the daily workload and was therefore not always optimal. In one ICU, data collection had to be performed by the principal investigator and the two medical assistants. This time-consuming work led to mixed team's feelings about the trial.

Turnover

Two ICUs had a high staff turnover, about 25 to 30 % per year. Since a learning period is necessary to newly arrived professionals to acquire resuscitation gestures and procedures, this may have impacted the time dedicated to training and fidelity to the strategy.

Opinion leader(s)’ attitudes towards the strategy

In all ICUs, the principal investigator could be considered as an opinion leader, answering questions from caregivers, and carefully ensuring that the protocol was applied by all professionals. This helped other professionals to take interest and invest in the study. However, two investigators expressed some reluctance regarding the strategy under scrutiny since it did not conform to their therapeutic habits. This might have impacted implementation of the strategy in their units by influencing other physicians' attitudes. Yet, the score of actual exposure to the strategy increased by more than 25 % between the control and the intervention periods in the two concerned ICUs (Table 3, centers B and C).

ICUs previous practices regarding fluid balance

While all physicians agreed on the importance of fluid balance monitoring in ICU, two of them acknowledged that limiting fluid overload was not always considered as a priority before the trial. Usual practices differed as shown by weekly average number of weigh-ins during the control period, which varied between two and five (Table 3).

Prescriptions habits regarding albumin also differed. One ICU used it as part of the treatment of liver failure or severe septic shock, but not as a standard filling procedure.

Other strategies were used by physicians to assess fluid balance in patients: in one ICU, cardiac ultrasonography was carried out almost systematically; two other ICUs frequently used input-output assessment before the trial, one of them being equipped with a particularly high-performance software, allowing automatic calculation of the input-output ratio based on prescription and information collected by nurses. In one non-computerized ICU, monitoring of the input-output balance was much more complex, and therefore not systematic.

Supplement 4. Results about study environment as a meso-contextual factor influencing POINCARE-2 strategy implementation

Scientific dynamic

Since most ICUs from teaching hospitals were already involved in numerous research protocols at the same time and benefited from dedicated research teams, scientific dynamic was more embedded in these ICUs than in ICUs from non-teaching hospitals.

Anticipated knowledge of the strategy

In two ICUs, some of the interviewed medical staff reported anticipated knowledge of the strategy during the control period. Yet, the score of actual exposure to the strategy during the control period remained low (under 40 %) in these two ICUs (Table 3, Centers C and H).

Study training

Training of medical investigators and research staff to the strategy was carried out by the coordination team at the beginning of the intervention period. Information was then passed on to other professionals by investigators, or research teams in teaching hospitals. Because it was difficult to bring all professionals together at once, it was not possible to organize a meeting dedicated to the study, but other media were used, such as displays in the break room, diffusion through the professional messaging system, or use of flyers. While physicians were mostly satisfied with the training provided by the coordination team, two nurses reported that they would have liked to receive more information about the hypotheses underpinning the trial.

Attitudes towards the research team (coordination and Clinical Research Associates (CRA))

Investigators stressed the essential part assumed by CRA who were in charge of data collection in teaching hospitals, which greatly relieved health professionals. The positive appreciation of the trial coordinating investigator by all interviewed physicians may also have strengthened adherence to the strategy in some ICUs.

One investigator mentioned that they would have appreciated to get more visits from the coordination team. Another one, as well as two medical assistants in charge of data collection emphasized their reactivity and availability.

Nurses and assistant nurses were rarely in contact with researchers, and one head nurse mentioned an overall lack of connection between health professionals and research teams, which sometimes led to a feeling of demotivation in non-medical staff.

Supplement 5. Results about health professionals as a micro-contextual factor influencing POINCARE-2 strategy implementation

Professional's attitudes towards hospital and ICU policies

This topic was raised by two ICU heads from non-teaching hospitals, who felt little support from their institution regarding research. This could lead to a feeling of isolation and demotivation towards research for medical teams in the concerned ICUs.

Clinical training and experience

Professional background was important to consider to better understand the perception on the strategy and its implementation. One investigator was a trained nephrologist, which could explain specific consideration for fluid balance before the trial, and his role as a lever to enhance implementation of the strategy in his ICU, with a score of actual exposure to the strategy above 60 % during the control period and 75 % during the intervention period (Table 3, Center B). An assistant nurse also worked in the logistics department of the ICU and was therefore particularly aware of equipment supply difficulties.

Research training and experience

Personal research background greatly varied depending on whether the ICU belonged to a teaching hospital or not. All professionals in teaching hospitals had already been involved in several research projects, and both investigators had graduate training in epidemiology. In the two ICUs from non-teaching hospitals however, the trial was frequently described by nurses as their first experience in research. Physicians, on the other hand, often had experience following a previous stay in a teaching hospital during their training.

Dedication and motivation towards research

All investigators were involved and concerned about protocol application. Interviewed physicians seemed to be ready to adapt their practices if the effectiveness of daily weighing was evidenced. In non-teaching hospitals, physicians were also motivated to take part in a study protocol to improve their ICU reputation through research and recruit more practitioners. But in three ICUs, investigators mentioned that some physicians did not comply with the strategy, which may have led to suboptimal application of the strategy, particularly during night or weekend shifts.

Nurses and assistant nurses mostly undervalued their own implication, while their input was recognized by head nurses and investigators. In one ICU, nurses could take the initiative to submit a POINCARE-2 follow-up sheet as soon as a new patient under mechanical ventilation

was admitted into the unit. Another nursing team developed its own tools to facilitate calculations of the input-output balance. However, while research value was largely appreciated by nurses, some reluctance was expressed due to material and logistical constraints, as stated above. Several nurses thus indicated that, even if daily weighing were proved effective, it would only be executed provided that more material resources are available.

Knowledge and attitudes towards fluid balance control

Physicians were all aware of the negative consequences of fluid overload in patients. One physician relied mainly on weight changes or the presence of edema and made extensive use of cardiac ultrasound to assess fluid overload. Another used input-output assessment, rather than weight, because of possible measurement errors. The benefit of albumin was perceived as uncertain, and its use was therefore mainly empirical before the trial implementation in most ICUs.
